# Control of Human Endometrial Stromal Cell Motility by PDGF-BB, HB-EGF and Trophoblast-Secreted Factors

**DOI:** 10.1371/journal.pone.0054336

**Published:** 2013-01-21

**Authors:** Maren Schwenke, Martin Knöfler, Philipp Velicky, Charlotte H. E. Weimar, Michelle Kruse, Annemarie Samalecos, Anja Wolf, Nick S. Macklon, Ana-Maria Bamberger, Birgit Gellersen

**Affiliations:** 1 Endokrinologikum Hamburg, Hamburg, Germany; 2 Department of Obstetrics and Fetal-Maternal Medicine, Reproductive Biology Unit, Medical University of Vienna, Vienna, Austria; 3 Laboratory of Neuroimmunology and Developmental Origins of Disease (NIDOD), University Medical Center Utrecht, Utrecht, The Netherlands; 4 Human Development and Health, Faculty of Medicine, University of Southampton, Princess Anne Hospital, Southampton, United Kingdom; 5 Endocrinology and Metabolism of Ageing, University Medical Center Hamburg-Eppendorf, Hamburg, Germany; Indiana University School of Medicine, United States of America

## Abstract

Human implantation involves extensive tissue remodeling at the fetal-maternal interface. It is becoming increasingly evident that not only trophoblast, but also decidualizing endometrial stromal cells are inherently motile and invasive, and likely contribute to the highly dynamic processes at the implantation site. The present study was undertaken to further characterize the mechanisms involved in the regulation of endometrial stromal cell motility and to identify trophoblast-derived factors that modulate migration. Among local growth factors known to be present at the time of implantation, heparin-binding epidermal growth factor-like growth factor (HB-EGF) triggered chemotaxis (directed locomotion), whereas platelet-derived growth factor (PDGF)-BB elicited both chemotaxis and chemokinesis (non-directed locomotion) of endometrial stromal cells. Supernatants of the trophoblast cell line AC-1M88 and of first trimester villous explant cultures stimulated chemotaxis but not chemokinesis. Proteome profiling for cytokines and angiogenesis factors revealed neither PDGF-BB nor HB-EGF in conditioned media from trophoblast cells or villous explants, while placental growth factor, vascular endothelial growth factor and PDGF-AA were identified as prominent secretory products. Among these, only PDGF-AA triggered endometrial stromal cell chemotaxis. Neutralization of PDGF-AA in trophoblast conditioned media, however, did not diminish chemoattractant activity, suggesting the presence of additional trophoblast-derived chemotactic factors. Pathway inhibitor studies revealed ERK1/2, PI3 kinase/Akt and p38 signaling as relevant for chemotactic motility, whereas chemokinesis depended primarily on PI3 kinase/Akt activation. Both chemotaxis and chemokinesis were stimulated upon inhibition of Rho-associated, coiled-coil containing protein kinase. The chemotactic response to trophoblast secretions was not blunted by inhibition of isolated signaling cascades, indicating activation of overlapping pathways in trophoblast-endometrial communication. In conclusion, trophoblast signals attract endometrial stromal cells, while PDGF-BB and HB-EGF, although not identified as trophoblast-derived, are local growth factors that may serve to fine-tune directed and non-directed migration at the implantation site.

## Introduction

In humans, blastocyst implantation and hemochorial placentation are highly invasive and dynamic processes. Diverse trophoblast populations arising from the trophectodermal shell of the blastocyst are in intimate crosstalk with the maternal decidua. The tips of anchoring villi harbor cell columns consisting of proliferating cytotrophoblast cells (CTB). These give rise to extravillous trophoblast cells (EVT) as they break through the syncytiotrophoblast covering the villi. Interstitial EVT invade the decidua as far as into the inner third of the myometrium, while endovascular EVT migrate into the uterine spiral arteries, displace the endothelial cells and remodel the vessels to establish increased blood flow into the intervillous space to support embryo development. Insufficient invasion is thought to contribute to severe pregnancy complications like preeclampsia and intra-uterine growth retardation [1]–[5]. The invasive capacity of EVT has been extensively studied, revealing a multitude of pathways involved in its regulation [6]. Chemotactic gradients are believed to direct invasion, and a balance of local proteases and protease inhibitors limits the extent of invasion in a temporal and spatial fashion [7]–[9].

Proper decidualization of endometrial stromal cells is critical to the establishment of pregnancy. The decidualization process is initiated in the mid-secretory phase of the menstrual cycle, independently of the presence of a blastocyst, and involves transformation of the elongated fibroblast-like endometrial stromal cells to larger cobblestone shaped secretory decidual cells [10]. Extensive reprogramming of the endometrial stromal cell gene expression profile upon decidualization results in altered cytoskeletal organization, extracellular matrix composition and adhesion, secretion of and responses to growth factors, cytokines and chemokines, and increased resistance to oxidative stress [11]–[13]. Downregulation of α-smooth muscle actin (α-SMA) causes a disruption of the cytoskeleton associated with morphological transformation [14]. Typical decidual marker genes are *PRL* and *IGFBP1* which are activated in response to cAMP and progesterone signaling and increased activity of the transcription factor forkhead box protein FOXO1 [15].

Decidualized cells deposit a dense extracellular matrix which poses a physical barrier to trophoblast invasion, yet the decidua with its resident leukocytes also provides a chemoattractant microenvironment promoting invasion [16]. Decidualization renders endometrial stromal cells more supportive to trophoblast expansion [17]. Impaired decidualization has been linked to defective embryo-maternal communication and recurrent pregnancy loss [18]. It is becoming increasingly clear that the decidua is more than a passive matrix for the implanting blastocyst, is able to sense embryo quality and may negate implantation of compromised embryos [19], [20]. Moreover, decidualized cells themselves are motile and invasive and are envisaged to actively support the profound tissue remodeling associated with implantation and placentation [21]–[23]. Cytoskeletal reorganization of decidualized endometrial stromal cells is regulated by Rho GTPases and supports invasion of cocultured blastocysts in an *in-vitro* implantation model [24], [25].

Cell migration is fundamental to implantation, embryogenesis, immune responses and wound healing. Locomotion can either be random (chemokinesis; triggered by a uniformly present stimulus) or directed (chemotaxis; following an external cue in a concentration gradient) [26]. Both processes have been observed in undifferentiated endometrial stromal cells in response to endocannabinoids and shown to involve activation of the PI3K and ERK1/2 pathways [27]. Endometrial stromal cells also mount a motile response to the angiogenic growth factor PDGF-BB, a phenomenon which may contribute to the regeneration of the endometrial functional layer after menstruation [28]. Another local growth factor with chemotactic activity is HB-EGF which promotes differentiation of trophoblast cells to the invasive phenotype [29], [30]. Conditional deletion of uterine HB-EGF in the mouse leads to impaired implantation, and an important role of this factor in embryo-uterine communication is also suggested in humans [31], [32]. However, the effect of HB-EGF on endometrial stromal cell motility has not been elucidated. Invasive trophoblast secretes numerous factors potentially controlling endometrial/decidual cell motility, however, these factors remain to be isolated.

Hence, the present study was undertaken to identify trophoblast-derived factors involved in the regulation of endometrial stromal cell random and directed motility, and to further characterize underlying signaling pathways.

## Materials and Methods

### Growth factors, cytokines, hormones, neutralizing antibodies, inhibitors

Human recombinant HB-EGF, insulin, 17*β*-estradiol (E2) and medroxyprogesterone acetate (MPA) were from Sigma-Aldrich (Deisenhofen, Germany), PDGF-AA, placental growth factor (PLGF-1), vascular endothelial growth factor (VEGF-165) from Peprotech (Hamburg, Germany), and PDGF-BB from Biomol (Hamburg, Germany). Neutralizing antibodies to HB-EGF (mouse monoclonal, clone 406316) and PDGF (goat polyclonal, recognizing PDGF-AA, -AB, -BB) were from R&D Systems (Abingdon, UK).

The following inhibitors were used: EGFR/ERBB2/ERBB4 (EGFR1/2/4) inhibitor (Calbiochem; Merck Chemicals, Darmstadt, Germany), AG1478 (tyrosine kinase inhibitor), Wortmannin (PI3 kinase inhibitor), SB202190 (p38 inhibitor) (Sigma), Y27632 (inhibitor of Rho-associated, coiled-coil containing protein kinase; ROCK), NSC23766 (Rac1 inhibitor) (Tocris, Bristol, UK), PD98059 (MEK1/2 inhibitor), and LY294002 (PI3 kinase inhibitor) (New England Biolabs, Frankfurt, Germany).

### Cell Culture

#### Primary human endometrial stromal cells (hESCs)

Hysterectomy specimens were obtained from premenopausal women (operated for benign indications) with no history of recurrent miscarriage and were taken randomly in the cycle. This study was approved by the Medical Review Ethics Committee University Medical Center Utrecht and the Central Committee on Research involving Human Subjects in The Netherlands (NL30143.000.09). Written informed consent was obtained from all participating subjects. Endometrial tissues were processed and hESCs isolated as previously described [22]. Cells were seeded in phenolred-free Dulbecco's modified Eagle medium (DMEM)/Ham's F12 (F12) supplemented with 1% amphotericin B (Sigma), 1% penicillin/streptomycin solution (Life Technologies, Paisley, U.K.) and 10% heat-inactivated fetal bovine serum (FCS). Individual hESC cultures were expanded to passage 2 and frozen in aliquots at −150°C.

Frozen hESCs were resuscitated and maintained in hESC medium: phenolred-free DMEM/F12 with 10% steroid-depleted dialysed FCS (PromoCell, Heidelberg, Germany), 100 U/ml penicillin, 100 μg/ml streptomycin, and supplemented with insulin (1 μg/ml) and E2 (1 nM). Decidualization was induced in minimal medium 1 (MM1-2%; phenolred-free DMEM/F12 supplemented with 2% steroid-depleted FCS and antibiotics) by 5 d treatment with 0.5 mM 8-Br-cAMP (Biolog, Bremen, Germany) and 1 µM MPA. Decidualization was assessed by RT-PCR for expression of the marker genes *PRL*, *IGFBP1* and *FOXO1*, and by measurement of PRL and IGFBP-1 in culture supernatants (Figure S1).

#### St-T1b and AC-1M88 cell lines

The telomerase-immortalized human endometrial stromal cell line, St-T1b, has been characterized previously [33]. Cells were maintained and decidualized as described for primary hESCs.

The human trophoblast cell line AC-1M88, a hybridoma of primary EVT from term placenta with a selectable mutant of the JEG-3 choriocarcinoma cell line [34], [35], was maintained in DMEM/F12 with 10% complete FCS, 100 U/ml penicillin, and 100 μg/ml streptomycin. For preparation of trophoblast conditioned medium (TCM), AC-1M88 cells were plated at a density of 4.2×10^6^ cells per 12 ml of culture medium in T25 cell culture flasks. The next day, medium was replaced by MM1-10% (composition as MM1-2% but containing 10% dialysed FCS) or Opti-MEM reduced serum media (Life Technologies), depending on the intended use of the conditioned media. The supernatant was harvested 24 h later.

### Generation of Conditioned Medium from Villous Explant Cultures

Placental tissues of early pregnancy (between 6 and 11 weeks of gestation) were obtained from legal abortions of uncomplicated pregnancies. Utilization of tissues was approved by the ethical committee of the Medical University of Vienna requiring informed patient consent.

Placental villous explants of 2–3 mm in size were carefully dissected from first-trimester placentae in pre-warmed culture medium (serum-free DMEM/F12 supplemented with gentamycin [50 µg/ml; Life Technologies, Paisley, U.K.]). Subsequently, explants were incubated floating in culture medium overnight at 37°C, 5% CO_2_. The next day, rat-tail collagen Type I (BD Biosciences, Bedford, MA), supplemented with 10x DMEM/low glucose (1∶10; Sigma) and 7.5% sodium bicarbonate (1∶5; Sigma), was poured to a 4 cm tissue culture dish. After formation of gels (30 min at 37°C, followed by 10 min equilibration in culture medium), 25 explants were placed on top of each humid collagen-field and incubated for 5 h at 37°C to allow anchorage. Subsequently, explants were supplemented with 4 ml culture medium and incubated for 3–4 days at 37°C and 5% CO_2_ to allow outgrowth of EVT. Upon harvesting, pooling and centrifugation (5 min, 2000 *g*), the supernatant was snap frozen in liquid nitrogen and stored at −80°C. These supernatants are designated as villous explant conditioned media (VECM). As a control, culture medium alone was incubated and stored as described above (VECM-Co).

### Proteome Profiling Arrays

For preparation of conditioned medium from trophoblast or endometrial stromal cell lines, AC-1M88 cells or pre-decidualized St-T1b cells were suspended in MM1-2% and plated at a density of 1.2×10^6^ cells/well in 6-well plates. The next day, medium was replaced by 1.1 ml fresh MM1-2% and the supernatant harvested 24 h later. Aliquots were stored at −80°C for proteome analysis. Proteome Profiler Array Kits (Human Cytokine Array Panel A, or Human Angiogenesis Array) were purchased from R&D Systems and applied according to the manufacturer's instructions, using 1 ml conditioned medium (from villous explant cultures, AC-1M88 cells, or decidualized St-T1b cells) per membrane. The cytokine array membrane carries capture antibodies to 36 factors, the angiogenesis array membrane carries antibodies to 55 factors, each spotted in duplicate. The samples were mixed with a cocktail of biotinylated detection antibodies, allowed to bind to the membranes, followed by several washes and incubation with streptavidin-HRP and chemiluminescence reagent. After exposure to film, signal intensities were quantified using ImageJ software. Values for integrated densities of each spot were corrected for background signal and normalized to positive control spots contained on each membrane. After graphical plotting in Excel, the X-axis was divided into three sections and values within the lower, middle or upper third of the range were classified as weak (+), medium (++) or strong (+++) signal, respectively.

### Chemotactic Migration Assay

Directed migration was monitored by transwell migration assay, using non-coated cell culture inserts with 8 µm pores for 24-well recipient plates (BD Biosciences, Heidelberg, Germany). St-T1b cells or hESCs were plated at a density of 3×10^4^ cells/insert in 350 µl Opti-MEM. The lower reservoir received 750 µl MM1-10% without or with supposed chemoattractive factors, TCM generated in MM1-10%, VECM, or VECM-Co. If inhibitors were included in the assay, cells were suspended in 100 µl Opti-MEM, allowed to attach to the insert for 3 h, then 250 µl of inhibitor in Opti-MEM, diluted to achieve the intended final concentration, was added and preincubated with the cells for 1 h. Then chemoattractant in 750 µl MM1-10% was added to the lower reservoir. Each treatment was performed in quadruplicate wells. Migration was allowed to proceed for 18 h. Cells were fixed in methanol for 2.5 min, followed by staining in solutions I and II from the Diff-Quik Staining Kit (Siemens Healthcare Diagnostics, Eschborn, Germany) for 2.5 min each. From three of the four inserts, the non-migrated cells from the upper side of the membrane were wiped off with a cotton swab, while from the forth insert, the migrated cells on the lower side were removed. On each membrane, cells were counted in six randomly selected visual fields (10× objective) and the mean number of cells per visual field (N) was determined. Numbers of cells on the upper side (N_U_), added to numbers of cells on the lower side (N_L_, mean from 3 membranes) yielded the total cell number (N_T_) per visual field. The percentage of migrated cells, designated as the motility index, was calculated as follows: motility index  =  N_L_/N_T_ ×100. This procedure takes into account that decidualization status or addition of growth factors or inhibitors might affect viability or proliferation rate during the migration period.

### Chemokinetic Migration Assay

Non-directed migration was determined using the Oris^TM^ Cell Migration Assay (AMS Biotechnology, Abingdon, U.K.). Principally, a central silicon stopper is inserted into each well of a black 96-well culture plate with clear flat bottoms. The stopper prevents cell attachment following cell seeding, creating a cell-free central migration zone. St-T1b cells suspended in 100 µl MM1-10% (4×10^4^ non-decidualized or 5×10^4^ decidualized cells) were plated in every well around the stoppers. Cells were allowed to attach for 4 h, then the stoppers were removed and the monolayers washed free of debris. One-hundred µl fresh MM1-10% without or with factors, or TCM generated in MM1-10%, were added. For inhibitor studies, the monolayers were preincubated with 100 µl MM1-10% supplemented with the inhibitors for 1 h after stopper removal. Subsequently, 100 µl MM1-10% with factors were added to achieve the desired final factor concentration. Cells were permitted to migrate into the central detection zone for 18 h. Each treatment was done in quadruplicate wells. Quantification of cell migration was not performed according to the manufacturer's instructions but as detailed in the Supporting Information (Protocol S1).

### Western Blotting

Whole cell extracts were prepared in RIPA buffer (0.1% sodium dodecylsulfate, 1% Triton X-100, 1% sodium deoxycholate, 150 mM NaCl, 1 mM EDTA, 10 mM Tris-HCl, pH 7.4) with complete protease inhibitors (Roche Applied Science, Mannheim, Germany). Cells for short-term stimulations were plated in 12-well plates, starved overnight in Opti-MEM, stimulated with factors diluted in Opti-MEM (5–60 min, 24 h), and harvested in 60 µl Quick Extract Buffer (QEB) heated to 85°C (2% sodium dodecylsulfate, 10% glycerol, 0.1% bromophenol blue, 5% *β*-mercaptoethanol, 50 mM Tris-HCl, pH 6.8). RIPA extracts (10–30 µg protein/lane, mixed with loading buffer) or QEB extracts (15 µl/lane) were electrophoresed on 10% or 4–12% SDS-polyacrylamide gels (NuPage Bis-Tris; Invitrogen) and transferred by tank-blotting onto polyvinylidene difluoride Immobilon membranes (Millipore, Eschborn, Germany) as detailed previously [33]. Immunodetection was performed with the enhanced chemiluminescence system (SuperSignal; Pierce, Bonn, Germany). Primary antibodies were directed to: GAPDH (clone 6C5, 1∶10,000; HyTest, Turku, Finland), α-SMA (clone 1A4, 1∶1000; Dako, Hamburg, Germany), p38, phospho-p38, ERK1/2, Akt, phospho-Akt(Ser473), MLC2, phospho-MLC2(Ser19) (all 1∶1000; New England Biolabs), phospho-ERK1/2 (1∶2000; New England Biolabs), HER4 (clone E200, 1∶5000), Nur77 (clone EPR3209, 1∶2000) (both from Epitomics, Burlingame, CA, USA), and HER1 (either: clone D38B1, 1∶5000; New England Biolabs, or: #sc-03, 1∶1000; Santa Cruz Biotechnology, Heidelberg, Germany). Secondary antibodies (HRP-conjugated anti-mouse or anti-rabbit IgG) were from Dianova (Hamburg, Germany).

### Statistical Analysis

Data were analyzed by ANOVA followed by Tukey or Dunnett *post-hoc* test, using GraphPad Prism software. Unless indicated otherwise, each experiment was repeated at least three times and figures reflect representative experiments with means ± SD of n = 3–4 replicates.

## Results

### Chemotactic response of endometrial stromal cells to PDGF-BB, HB-EGF, and conditioned medium from the trophoblast cell line AC-1M88

**Figure 1:**
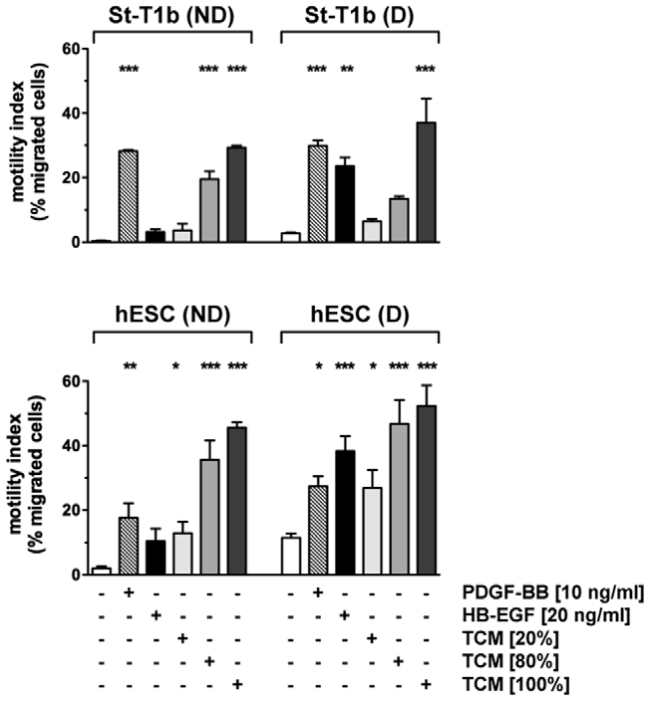
Chemotactic response of St-T1b cells or primary hESCs to PDGF-BB, HB-EGF and trophoblast conditioned medium. St-T1b cells (*upper panel*) or hESC (*lower panel*), non-decidualized (ND) or decidualized (D) by 5 d treatment with 8-Br-cAMP/MPA, were subjected to transwell migration assay. The bottom reservoir contained MM1-10% (controls), PDGF-BB, HB-EGF or conditioned medium (TCM) from the trophoblast cell line AC-1M88 undiluted (100%) or diluted to 80% or 20%. The motility index designates the percentage of migrated cells relative to the total cell number (non-migrated plus migrated) at the end of the 18 h migration period. Shown are results representative of 3 similar experiments (means± SD, n = 3). Data were analyzed by ANOVA and Dunnett *post-hoc* test. *, *P*<0.05; **, *P*<0.01; *** *P*<0.001 compared to the respective control within the ND or D groups.

**Figure 2:**
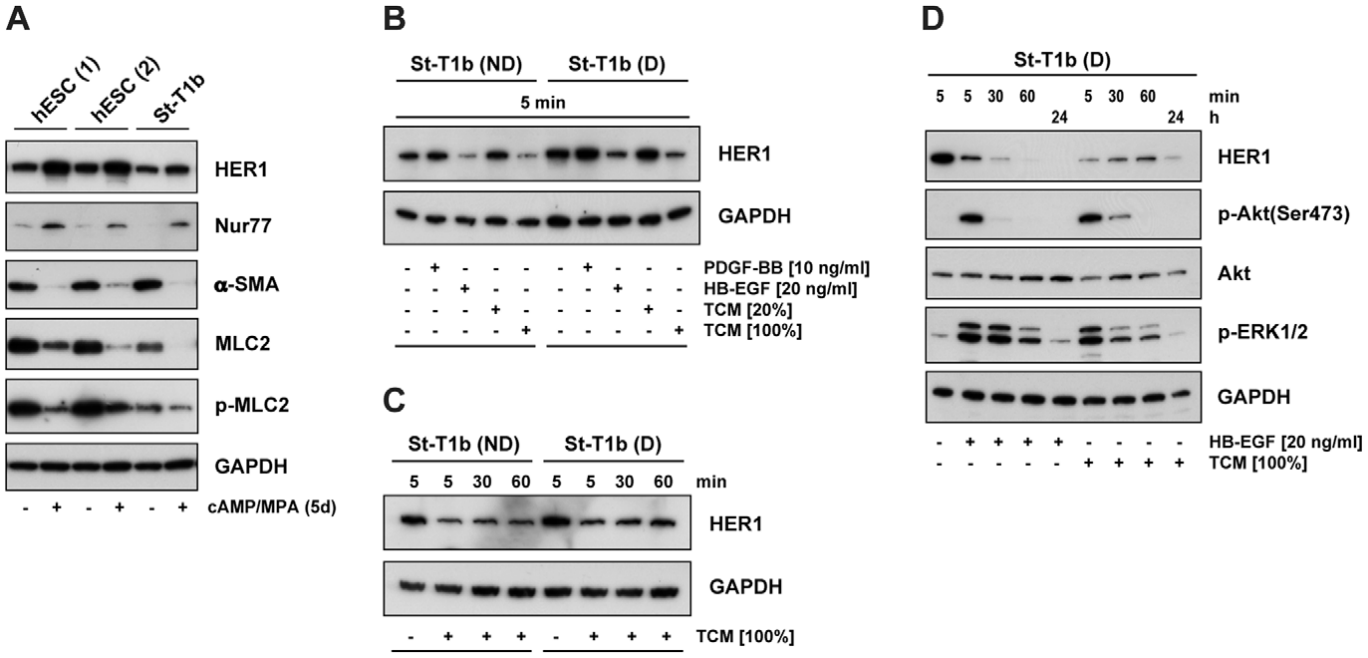
Expression and regulation of HER1 in St-T1b cells and hESCs. (**A**) Effect of decidualization on HER1 expression. Whole cell extracts from two individual hESC preparations or from St-T1b cells, non-decidualized (−) or decidualized (+), were immunoblotted for HER1 (antibody from New England Biolabs), and for GAPDH as a loading control. In addition, the levels of Nur77, α-SMA, MLC2 and phospho-MLC2 were analyzed. (**B, C, D**) Time-dependent downregulation of HER1 in response to HB-EGF or TCM. Non-decidualized (ND) or decidualized (D) St-T1b cells, maintained in Opti-MEM, were incubated with PDGF-BB, HB-EGF or TCM (20 or 100%) for 5 min (**B**) or 5, 30 and 60 min (**C**) and subjected to Western Blot analysis for HER1 (antibody from Santa Cruz). Decidualized St-T1b cells were stimulated with HB-EGF or TCM (100%) for 5, 30 and 60 min or 24 h **(D)** and analyzed for HER1 by Western blotting (antibody from Santa Cruz), followed by immunoblotting for phospho-ERK1/2 and phosphorylated (Ser473) and total Akt.

In an initial experiment, we investigated the chemotactic response of the immortalized endometrial stromal cell line St-T1b to PDGF-BB, HB-EGF and trophoblast conditioned medium (TCM), harvested from the EVT cell line, AC-1M88 (Figure 1). TCM stimulated chemotaxis of St-T1b cells in a dose-dependent fashion, and PDGF-BB also elicited a marked migratory response, both in non-decidualized and decidualized cells. In contrast, HB-EGF only caused a significant response in decidualized St-T1b cells. Comparable results were seen with primary hESCs (Figure 1). Because we consistently observed a stronger migratory effect of HB-EGF on decidualized compared to undifferentiated endometrial stromal cells, we monitored expression of HB-EGF receptors (HER1, HER4) by Western blot analysis (Figure 2A). Both in hESCs and in St-T1b cells, HER1 expression was elevated upon decidualization. HER4 was not detectable (data not shown; function of the HER4 antibody was confirmed by detecting a strong positive signal in the human breast cancer cell line, T47D). As a marker of decidualization, we observed upregulation of the orphan nuclear receptor Nur77, which has also recently been identified as a modulator of mesenchymal stromal cell migration [36], [37]. As expected, α-SMA was downregulated in conjunction with decidualization [14], as was another cytoskeletal protein, MLC2, along with its phosphorylated form.

We then investigated whether HB-EGF might be a chemoattractive component of TCM. Incubation of St-T1b cells with HB-EGF resulted in rapid downregulation of HER1 within 5 min, with no recovery over 60 min (Figure 2B,C,D). Incubation with TCM, at the higher dose which had proven chemotactic activity, also caused loss of HER1 with similar kinetics. These responses were independent of decidualization. PDGF-BB was without effect on HER1 expression (Figure 2B). In decidualized St-T1b cells, HB-EGF and TCM both activated the Akt and ERK1/2 pathways, again with comparable kinetics (Figure 2D). Phosphorylation of Akt (Ser473) occurred within 5 min and was nearly gone by 30 min, whereas phosphorylation of ERK1/2 was also maximal at 5 min but remained elevated for at least 60 min in response to both stimuli. Activation of signaling subsided within 24 h, while HER1 protein levels remained suppressed.

**Figure 3:**
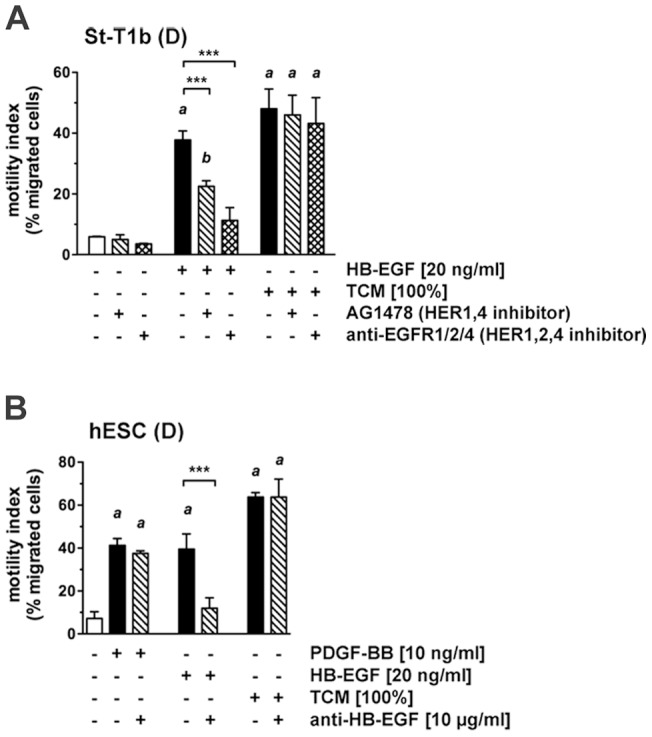
Effect of EGF receptor antagonists or HB-EGF neutralizing antibody on chemotactic migration of St-T1b cells or hESCs. (**A**) Decidualized St-T1b cells were preincubated in transwell migration inserts with the tyrosine kinase inhibitor AG1478 (100 nM) or the EGFR1/2/4 (HER1/HER2/HER4) inhibitor (10 nM) for 1 h before addition of HB-EGF or TCM to the lower compartment for another 18 h. The percentage of migrated cells relative to the total cell number is given as the motility index (means±SD, n = 3). Results were analyzed by ANOVA and Dunnett or Tukey test. ***, *P*<0.001 compared to the respective control without inhibitor; *a*, *P*<0.001, *b*, *P*<0.01 compared to non-stimulated cells without inhibitor (open column). (**B**) Decidualized hESCs were subjected to transwell migration assay with PDGF-BB, HB-EGF or TCM as the chemoattractant, in the absence or presence of a neutralizing antibody to HB-EGF. Motility indices (means±SD, n = 3) were analyzed by ANOVA and Tukey test. *a*, *P*<0.001 compared to the control without chemoattractant or antibody (white column); ***, *P*<0.001 compared between conditions without or with antibody.

The above findings strongly suggested the presence of HB-EGF, or a related HER1 ligand, in TCM. To further investigate this in a functional assay, chemotactic migration towards TCM was assessed in the absence or presence of the tyrosine kinase inhibitor AG1478, targeting HER1/HER4, or the EGFR/ERBB2/ERBB4 inhibitor, targeting HER1/HER2/HER4 (Figure 3A). While both substances significantly reduced St-T1b migration in response to HB-EGF, no effect on TCM-stimulated migration was seen. Lastly, a neutralizing antibody to HB-EGF was employed at a concentration sufficient to fully block chemotaxis towards HB-EGF (Figure 3B). However, neutralization of HB-EGF did not reduce TCM-stimulated migration. The bioneutralizing antibody did not inhibit migration non-specifically, as chemotaxis towards PDGF-BB was not affected.

Taken together, TCM is a powerful chemoattractant for endometrial stromal cells. It contains a component that interacts with HER1 and causes downregulation of the receptor. However, TCM-induced migration does not seem to require activation of HER1, HER2 or HER4, nor does it depend on the presence of HB-EGF.

### Chemotactic response of endometrial stromal cells to conditioned medium from first trimester villous explant cultures

**Figure 4:**
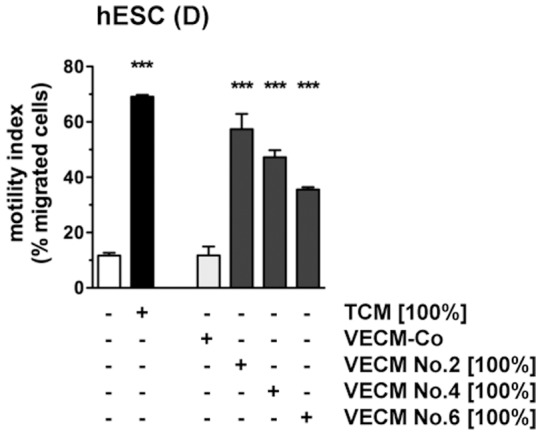
Chemotactic response of hESCs to conditioned medium from first trimester villous explant cultures. Decidualized hESCs were subjected to transwell migration assay using TCM, or supernatants from three individual first trimester villous explant cultures (VECM), as the chemoattractant (undiluted). The control medium for TCM treatment was MM1-10%, that for VECM was serum-free medium incubated with collagen as detailed in Materials & Methods (VECM-Co). Motility indices represent means±SD (n = 3) and were analyzed by ANOVA and Tukey test. ***, *P*<0.001 compared to the respective control.

Next, we wished to study the endometrial stromal cell migratory response to the secretions of primary trophoblast cells. Villous explant conditioned media (VECM) from villi of three individual placentae were employed in transwell migration assays with decidualized hESCs (Figure 4). All three preparations significantly stimulated chemotaxis over that seen with serum-free control medium (VECM-Co). The stimulatory activity of the most potent VECM was in the range of that seen with undiluted TCM from AC-1M88 cells. Upon dilution, activity of the villous explant samples was lost (data not shown), suggesting that concentration of active components was at the lower limit.

### Proteome profiling of secretions from AC-1M88 trophoblast cells and first trimester villous explant cultures

In an attempt to identify candidate chemoattractive constituents, TCM (from AC-1M88 cells) and VECM from two individual villous explant cultures were subjected to proteome profiling for cytokines and angiogenesis factors. To identify cell- or tissue-specific secretions, the TCM and VECM profiles were contrasted to the profile of decidualized St-T1b cells (Table 1). In addition, proteome profiles were compared to gene expression profiles previously generated from isolated EVT and CTB primary trophoblast populations [38]. While fewer factors were detected in AC-1M88 supernatants than in villous supernatants, almost all factors found in AC-1M88 supernatants were also present in villous supernatants. Moreover, expression of these factors had also been identified at the transcript level in purified EVT, supporting the cellular origin of the AC-1M88 cell line. Only coagulation factor III (tissue factor; TF) and tissue inhibitor of metalloproteinases 4 (TIMP4) were detected in AC-1M88 supernatant but were neither present in VECM nor in the transcript profiles of EVT or CTB. It should to be noted that a number of cytokines and growth factors, including amphiregulin (AREG), endothelin-1 (ET-1), hepatocyte growth factor (HGF), or CXCL4, were picked up in the proteome profile of villous explant supernatants while their expression had not been found at the transcript level in purified EVT or CTB by Bilban *et al* [38] (Table 1). This is underpinned by the absence of these mRNAs in an independent genome-wide expression profiling of CTB and EVT reported by Apps *et al* [39].

**Table 1:** Proteome profiling for cytokines and angiogenesis factors in conditioned medium from AC-1M88 human trophoblast cells, two individual first trimester villous explant cultures, and the St-T1b human endometrial stromal cell line after decidualizing treatment.

	Proteome Profiler Array a				Microarray *^b^*	Proteome Profiler Array a
Protein Name	AC-1M88	Villous Explant No. 2	Villous Explant No. 6	Gene Name	mRNA detected in	St-T1b (decidualized)
Angiogenin		+		ANG	( )	++
Angiopoietin-2		+		ANGPT2	EVT±, CTB+	
Amphiregulin		++	+	AREG	( )	
CCL2 (MCP-1)				CCL2	( )	+++
CD105 (Endoglin)	+	++	+	ENG	EVT+, CTB+	
CD154 (CD40 Ligand)				CD40LG	( )	+
CD26 (DPPIV)	+	+++	+++	DPP4	EVT+, CTB+	+
Coagulation Factor III (TF)	+			F3	( )	
CSF-2 (GM-CSF)				CSF2	( )	+
CXCL1 (GROα)		++	+	CXCL1	EVT±, CTB±	++
CXCL4		++		PF4	( )	
CXCL16		++	+	CXCL16	*not represented*	
Endostatin		+	+	COL18A1 *cleavage product*	( )	
ET-1 *^c^*		++	++	EDN1	( )	+
HGF		++	+	HGF	( )	+
IFN-γ				IFNG	( )	+
IGFBP-1		+++	+	IGFBP1	CTB±	
IGFBP-2		+++	++	IGFBP2	EVT±	
IGFBP-3		+++	+++	IGFBP3	EVT+, CTB+	+++
IL-1ra				IL1RN	( )	+
IL-6		+++	+++	IL6	EVT±, CTB+	+
IL-8 (CXCL8)		+++	+++	IL8	CTB±	+++
IL-23				IL23A *alpha-subunit*	( )	+
Leptin		+++	+++	LEP	EVT+, CTB+	
MIF	+++	++	+	GLIF, MMIF	EVT+, CTB+	+++
MMP-9	++	++	+	MMP9	EVT+, CTB+	++
PAI-1 (Serpin E1)	+++	+++	+++	SERPINE1	EVT+, CTB+	+++
PDGF-AA	+++	+		PDGFA	EVT±, CTB±	
Persephin		+		PSPN	( )	
PEDF (Serpin F1)	+	+	+	SERPINF1	EVT+, CTB+	
PLGF (Placental GF)	+++	++	++	PGF	EVT+, CTB+	
PRL				PRL	( )	+
Prokineticin-1		++	+	PROK1	*not represented*	
PTX3/TSG-14		+	+	PTX3	( )	+
TGF-β1 (LAP)	++	+		TGFB1 *cleavage product*	EVT+, CTB+	
Thymidine Phosphorylase		+		TYMP	EVT±, CTB±	
TIMP-1	+++	+++	+++	TIMP1	EVT+, CTB+	+++
TIMP-4	+			TIMP4	( )	
TSP-1		+		THBS1	CTB±	+
uPA		+		PLAU	EVT±, CTB±	
VEGF	+++	+	+	VEGFA	EVT+, CTB+	+++

a Proteome profiling: +, low; ++, medium; +++, strong signal intensity on Proteome Profiler Array membranes (cytokines and angiogenesis factors). Proteome profiles were compared to ***^b^*** Microarray mRNA expression profiles of EVT and villous CTB as reported in http://www.ncbi.nlm.nih.gov/geoprofiles?term=GDS3523 [38].

EVT+, invasive extravillous trophoblast, all 6 mRNA pools gave positive signal; CTB+, non-invasive villous cytotrophoblast, all 5 mRNA pools gave positive signal; EVT±, not all mRNA pools out of 6 gave positive signal; CTB±, not all mRNA pools out of 5 gave positive signal; ( ), not detected in EVT or CTB. ***^c^*** ET-1 protein was also detected in villous explant control media (+) and may therefore not be a specific explant product. No signal was obtained in any of the cell types for the following factors represented on the Proteome Profiler Array membranes for cytokines or angiogenesis factors: Activin-1, ADAMTS-1, Angiopoietin-1, Angiostatin, Artemin, CFS-3 (G-CSF), EGF, FGF-1, FGF-2, FGF-4, FGF-7, GDNF, C5/C5a, CCL1, CXCL10, CXCL11, CD54 (sICAM-1), CXCL12, HB-EGF, IL-1α, IL-1β, IL-2, IL-4, IL-5, IL-10, IL-12, IL-13, IL-16, IL-17, IL-27, IL-32α, MIP-1α, MIP-1β, MMP-8, NRG1-β1, PDGF-AB, PDGF-BB, RANTES (CCL5), Serpin B5 (Maspin), TNF-α, sTREM-1, TSP-2, Vasohibin, VEGF-C.

Numerous factors identified in the VECM proteome profile, but not in AC-1M88 cells, appear to be of CTB origin, e.g. IGFBP-1, IL-8 and TSP-1. IL-8 and TSP-1, in turn, were also products of decidualized St-T1b cells and support their decidualization status, as does the cell-specific expression of PRL. Among the prevalent products common to AC-1M88 cells, villous explants and St-T1b cells were macrophage migration inhibitory factor (MIF), matrix metalloproteinase 9 (MMP-9), plasminogen activator inhibitor (PAI-1), TIMP-1 and VEGF. Notably, neither PDGF-BB nor HB-EGF were detectable in any of the samples.

For the ensuing experiments, we focused on two proteins that were present in trophoblast cells and villous explants but absent from St-T1b cells, namely PDGF-AA and placental growth factor (PLGF), and on the ubiquitous factor VEGF.

### Chemotactic response of endometrial stromal cells to PDGF-AA, PLGF, and VEGF

**Figure 5:**
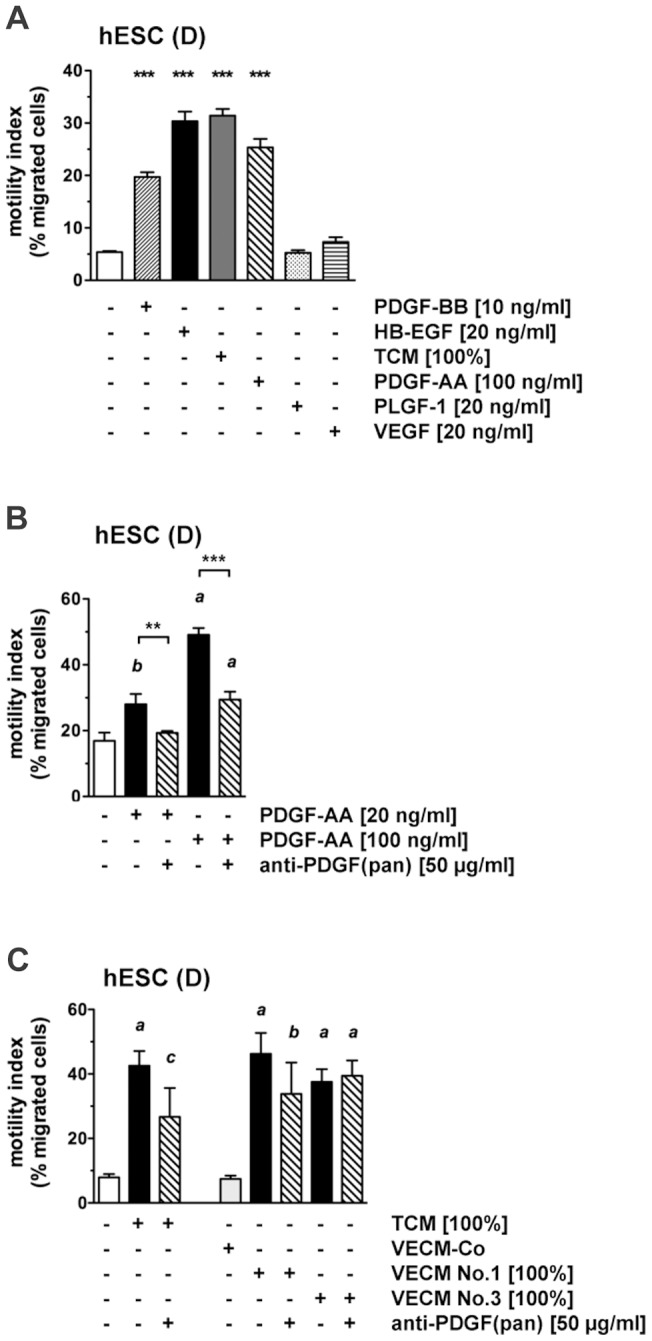
Chemotactic response of hESCs to trophoblast secretory products identified by proteome profiling. (**A**) Decidualized hESCs were analyzed in transwell migration assay in response to PDGF-BB, HB-EGF, TCM, PDGF-AA, PLGF-1 or VEGF-165. Motility indices are shown as means±SD (n = 3), and were analyzed by ANOVA and Dunnett test. ***, *P*<0.001 compared to the control without chemoattractant. (**B, C**) Effect of neutralization of PDGF activity. Decidualized hESCs were subjected to transwell migration assay with two different doses of PDGF-AA (**B**) or with TCM and two individual VECM preparations (**C**) in the absence or presence of a neutralizing antibody to PDGF-AA/-AB/-BB (pan). Motility indices are shown as means±SD (n = 3) and were analyzed by ANOVA and Dunnett or Tukey test. ***, *P*<0.001; **, *P*<0.01 in the absence vs. presence of antibody. *a*, *P*<0.001; *b*, *P*<0.01; *c*, *P*<0.05 compared to the respective control without stimulation or antibody (white or light grey columns).

Transwell migration of decidualized hESCs was assessed in response to the trophoblast-specific secretory products, PDGF-AA and PLGF, and to VEGF which was abundantly secreted by all cell types (Figure 5A). PDGF-AA (at 100 ng/ml) stimulated migration to an extent comparable to that of PDGF-BB (10 ng/ml), HB-EGF (20 ng/ml), or undiluted TCM. PLGF and VEGF (20 ng/ml) did not induce migration. Further experiments using up to 100 ng/ml of these factors also yielded no response (data not shown).

A neutralizing PDGF antibody, recognizing the PDGF-BB, -AB and -AA dimers, was added at a dose sufficient to inhibit migration stimulated by PDGF-AA (100 ng/ml) (Figure 5B,C). This antibody did however not antagonize chemotaxis driven by TCM or VECM, indicating that the chemotactic activity of PDGF-AA in trophoblast secretions is redundant.

### Chemokinetic response of endometrial stromal cells to PDGF-BB, HB-EGF, TCM and PLGF

**Figure 6:**
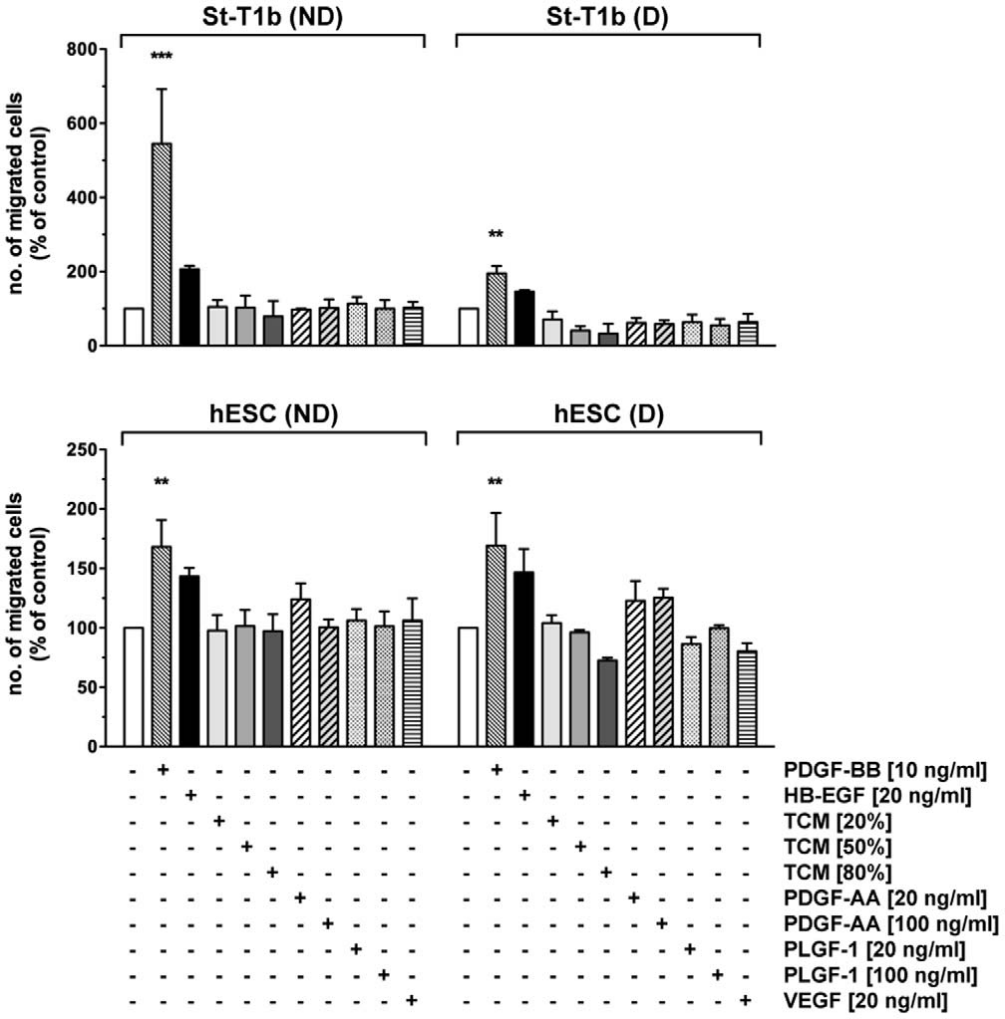
Chemokinetic response of St-T1b cells or primary hESCs to PDGF-BB, HB-EGF, TCM and trophoblast secretory products identified by proteome profiling. St-T1b cells (*upper panel*) or hESC (*lower panel*), non-decidualized (ND) or decidualized (D), were subjected to Oris migration assay to monitor random motility, in the presence of PDGF-BB, HB-EGF, TCM, PDGF-AA, PLGF-1, or VEGF-165 at the indicated concentrations. Numbers of cells that had migrated into the detection zone after 18 h incubation were determined and normalized to unstimulated controls within each ND or D group. Shown are the means±SEM of n = 3 independent experiments. Results were analyzed by ANOVA and Dunnett test within each group. ***, *P*<0.001; **, *P*<0.01 compared to untreated controls (white columns).

All factors tested in transwell migration assays, assessing chemotaxis in response to a concentration gradient, were also applied in an Oris migration assay to monitor random migration, i.e. chemokinesis, in response to a homogeneously dissolved factor. Only PDGF-BB significantly stimulated motility in this setting, in non-decidualized as well as in decidualized St-T1b or primary hESCs (Figure 6). In marked contrast to their chemoattractant activity, TCM, HB-EGF or PDGF-AA failed to enhance chemokinesis. PLGF and VEGF were again without effect.

### Elucidation of signaling pathways involved in regulation of chemotaxis or chemokinesis

**Figure 7:**
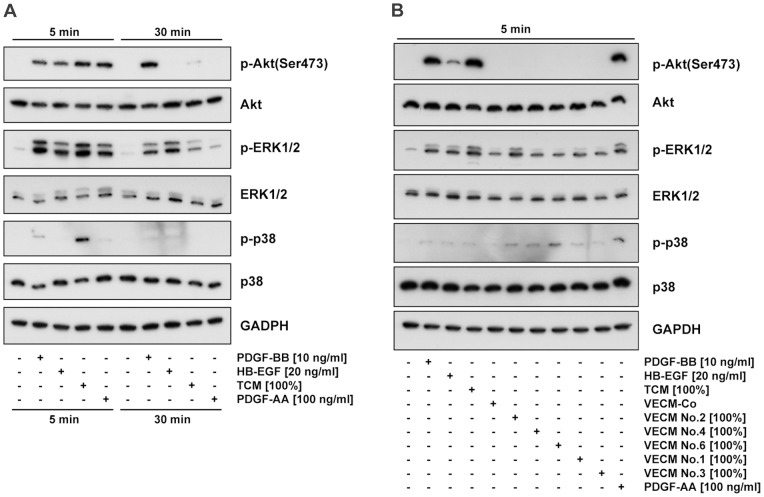
Signaling pathways activated by PDGF-AA, PDGF-BB, HB-EGF, TCM, or VECM. (**A**) Decidualized St-T1b were starved in Opti-MEM and then treated for 5 or 30 min with PDGF-BB, HB-EGF, TCM or PDGF-AA. Levels of phosphorylated (p-) or total ERK1/2, Akt, and p38 were determined by Western blotting. (**B**) Decidualized hESC were starved in Opti-MEM and then treated for 5 min with PDGF-BB, HB-EGF, TCM, 5 individual VECM preparations, villous explant control medium (VECM-Co), or PDGF-AA. Western blotting was performed as above.

The previous experiments revealed two groups of compounds: those which exclusively functioned as chemoattractants (HB-EGF, TCM, PDGF-AA) versus PDGF-BB which acted both as a chemoattractant and as a stimulus for random motility. We wondered if distinct signaling pathways were utilized by either group of stimulants. All four compounds yielded a phosphorylation of Akt and ERK1/2 in St-T1b cells and hESCs within 5 min (Figure 7). Phosphorylation of p38 was faint and not consistently seen. Activation of ERK1/2 in response to all four stimuli was maintained over 30 min. Sustained activation of Akt was only elicited with PDGF-BB while the responses to HB-EGF, TCM or PDGF-AA returned to control level within 30 min. VECM only yielded a very weak activation of ERK1/2 and p38 compared to treatment with villous explant control medium (Figure 7 B).

**Figure 8:**
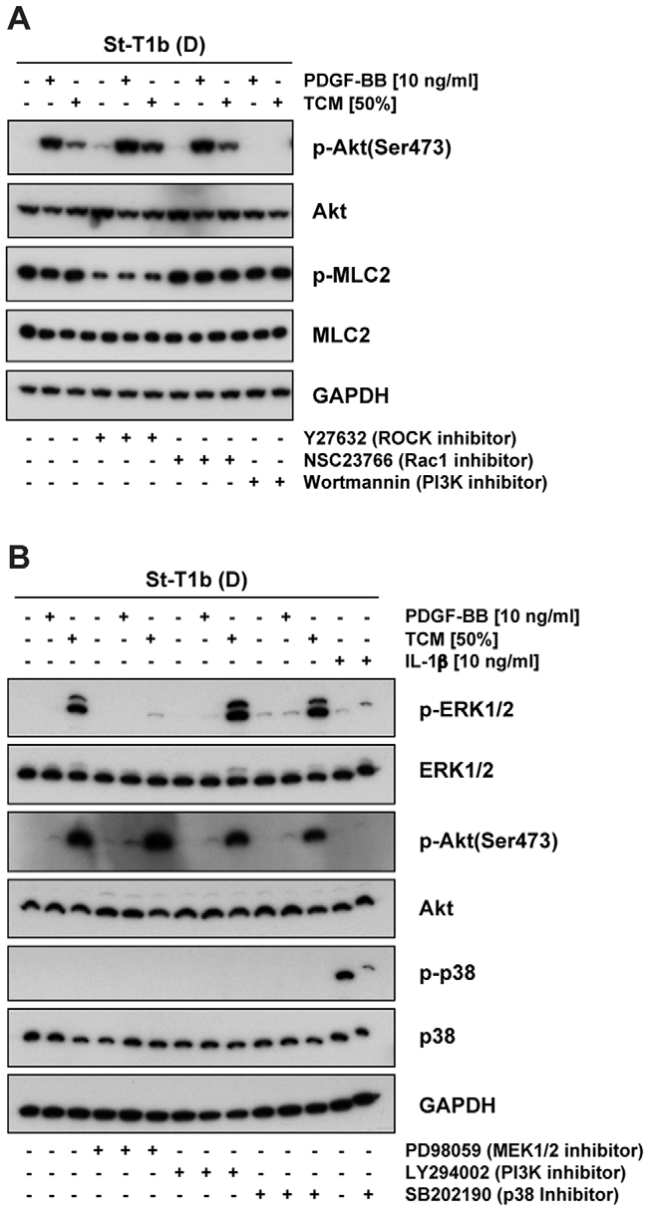
Functional validation of pathway inhibitors. Decidualized St-T1b were preincubated for 1 h with Y27632 (100 µM), NSC23766 (50 µM), Wortmannin (200 nM) (**A**) or PD98059 (50 µM), LY294002 (10 µM), SB202190 (10 µM) (**B**), followed by 5 min stimulation with PDGF-BB, TCM or IL-1β. Control cultures received Opti-MEM. Total or phosphorylated (p-) Akt, p38, ERK1/2 and MLC2 were detected by Western blot analysis.

We then tested effectiveness and specificity of signaling pathway inhibitors by incubating decidualized St-T1b cells with inhibitors prior to 5 min stimulation with PDGF-BB, TCM or IL-1β (used as a robust stimulus for p38 activation) (Figure 8). SB202190 prevented phosphorylation of p38, the PI3K inhibitor Wortmannin ablated, and LY294002 blunted activation of Akt, the MEK1/2 inhibitor PD98059 abolished ERK1/2 phosphorylation, and ROCK inhibitor Y27632 reduced basal levels of phospho-MLC2. Neither Rac1 inhibitor NSC23766 nor any other inhibitor significantly interfered with other pathways within this short-term stimulation. Wortmannin was chosen as PI3K inhibitor for further experiments as it was more effective than LY294002.

**Figure 9:**
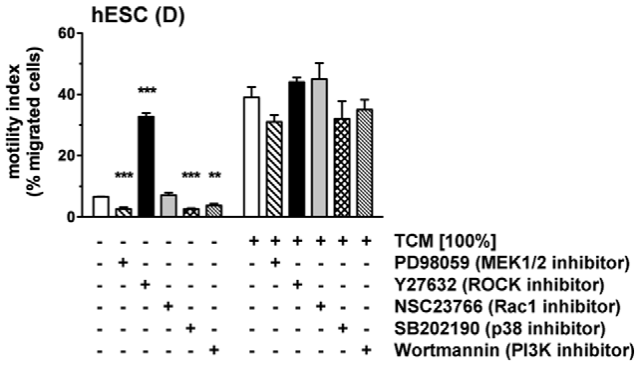
Effect of pathway inhibitors on chemotactic migration of hESCs. Decidualized hESCs in transwell migration inserts were preincubated with PD98059 (50 µM), Y27632 (100 µM), NSC23766 (50 µM), SB202190 (10 µM) or Wortmannin (200 nM) before the addition of TCM to the lower reservoir. Controls received MM1-10% instead of chemoattractant. Motility indices are means±SD (n = 3) and were analyzed by ANOVA and Dunnett test. **, *P*<0.01; ***, *P*<0.001 compared to cells without inhibitor in the group without chemoattractant (white column). No significant differences were seen within the group receiving TCM. Exemplary images of migrated cells are shown in Figure S2.

The above inhibitors were then added to decidualized hESCs to monitor the effect on basal or TCM-stimulated chemotaxis (Figure 9). None of the inhibitors significantly reduced migration towards TCM. However, basal migration was markedly affected; inhibition of ERK1/2, PI3K/Akt, or p38 signaling diminished, while the ROCK inhibitor vastly enhanced motility of hESCs. Microphotographs of migrated cells at the underside of the porous membrane are shown in Figure S2.

**Figure 10:**
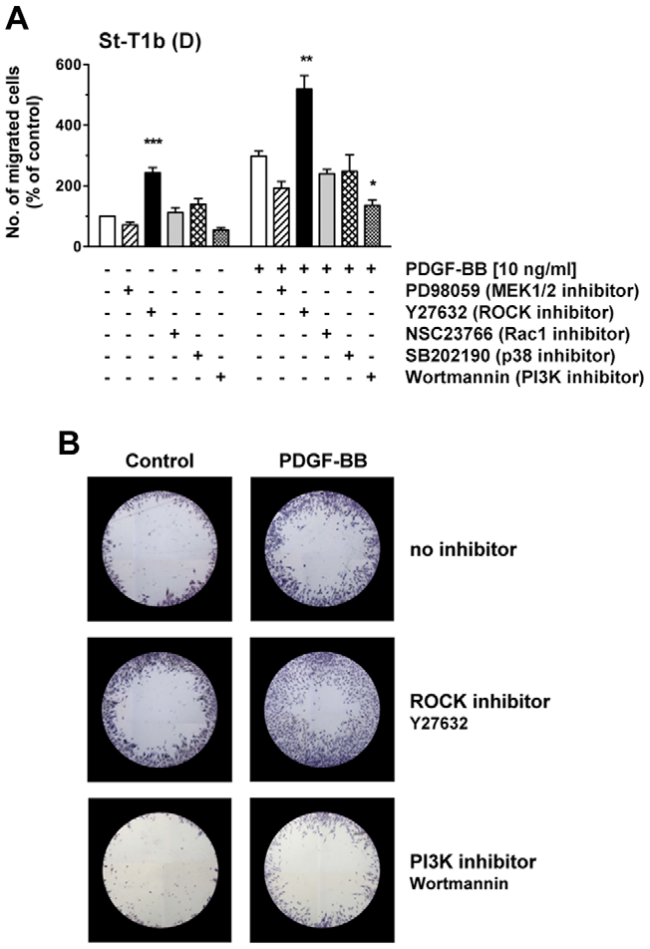
Effect of pathway inhibitors on chemokinetic motility of St-T1b cells. (**A**) Decidualized St-T1b cells were seeded in Oris migration plates and preincubated with inhibitors for 1 h: PD98059 (50 µM), Y27632 (100 µM), NSC23766 (50 µM), SB202190 (10 µM) or Wortmannin (200 nM). Then PDGF-BB was added to the indicated final concentration, or the equivalent volume of control medium, and cells allowed to migrate into the detection zone for 18 h. Numbers of migrated cells were normalized to the control in the absence of stimulus and inhibitor (left white column). Results represent the means±SEM of n = 3 independent experiments. Data were analyzed by ANOVA and Dunnett test within each group (unstimulated or treated with PDGF-BB) and revealed significant differences after inhibitor treatment compared to the respective controls without inhibitor: *, *P*<0.05; **, *P*<0.01; *** *P*<0.001. (**B**) Representative images of detection zones for the treatments evaluated in panel (**A**), showing cells that had migrated into the cell-free zone within 18 h. Cells were stained with Diff-Quik. Images of each detection zone were reassembled from 4 microphotographs taken with a 4× objective and graphically overlaid with the black mask.

Chemokinesis was then assessed by Oris migration assay under basal conditions, or under PDGF-BB stimulation (Figure 10A). PI3K inhibitor reduced PDGF-BB-stimulated migration, while ROCK inhibitor markedly enhanced both basal and stimulated migration of decidualized St-T1b cells. The appearance of migrated cells in the detection zone of the assay is illustrated in Figure 10B. ROCK inhibitor caused the cells to produce excessively long protrusions. This appearance clearly differed from that seen in the presence of PDGF-BB, although both compounds shared the ability to stimulate random and chemotactic migration (Figure S3).

Taken together, the ERK1/2, PI3K/Akt and p38 signaling pathways are involved in chemotactic motility, whereas chemokinesis requires primarily PI3K/Akt activation. ROCK signaling is inhibitory to both chemokinesis and chemotaxis.

## Discussion

Extensive crosstalk at the fetal-maternal interface orchestrates implantation and formation of the human placenta. It is widely accepted that trophoblast cells, particularly interstitial and endovascular EVT, follow chemoattractive gradients when invading the decidua and entering the maternal arteries [16]. The results of our present study provide novel evidence to the concept that decidual cells are likewise harnessed to migrate in response to local stimuli. We propose that decidualized endometrial stromal cells actively support the remodeling processes necessary for the establishment and maintenance of pregnancy.

Here we demonstrate a strong motile response of endometrial stromal cells to the angiogenic growth factor PDGF-BB. While we did not detect PDGF-BB in the supernatants of AC-1M88 trophoblast cells or first trimester villous explants, the factor has been found in uterine fluid taken from women in the receptive phase of the menstrual cycle [40]. PDGF-BB immunoreactivity has been localized to endometrial endothelial cells and vascular smooth muscle cells throughout the cycle, while endometrial stromal cells are positive for the receptor forms PDGF-Rα and -Rβ [41]. Undifferentiated hESCs have previously been shown to mount a variety of responses to PDGF-BB including enhanced contractility, migration, invasion and proliferation. This was suggested to aid in tissue repair after menstruation [42]. We show here that decidualized endometrial stromal cells likewise migrate upon exposure to PDGF-BB. Interestingly, of all factors investigated in the present study, PDGF-BB was the only one to induce not only chemotaxis but also chemokinesis. It may therefore serve to generally enhance motility of endometrial stromal cells without directing them towards a particular location. This was in marked contrast to the effect of PDGF-AA which solely functioned as a chemoattractant. We found PDGF-AA to be secreted by the trophoblast cell line AC-1M88 and by first trimester villous explants. The mRNA for PDGF-AA has also been demonstrated in trophectoderm from day 5 implantation-competent blastocysts while the corresponding receptor expression was seen in the receptive endometrium [43]. It is thus conceivable that blastocyst-derived PDGF-AA participates in attracting decidualizing endometrial stromal cells to the implantation site. Attracted cells then move around the implanting conceptus to engulf it, a process which we have recently documented by time-lapse video recording of decidualized hESCs cocultured with day 5 human embryos [22].

Another powerful chemoattractant was HB-EGF. It specifically stimulated migration of decidualized endometrial stromal cells, which may in part be due to upregulation of the HER1 receptor upon decidualization as we report here. While others have shown both HER1 and HER4 in cultured hESCs [32], [44], we only detected the HER1 form by Western blotting. Incubation with TCM or villous explant CM led to a profound and sustained downregulation of HER1 in endometrial stromal cells, very similar to that seen upon HB-EGF treatment. HER1 undergoes ligand-induced endocytosis and, depending on the ligand, recycling or degradation [45], [46]. The receptor is recycled upon binding of EGF, amphiregulin, TGFα or epiregulin, whereas HB-EGF and betacullin target HER1 for lysosomal degradation [47]. While HER1 in endometrial stromal cells was rapidly downregulated upon HB-EGF treatment, ERK1/2 activation persisted for at least one hour. Enhanced migration apparently results from HB-EGF triggering a signaling network the downstream components of which are largely independent of continued HER1 expression/activation. The effectors may include secondary response genes such as those encoding MMPs or cytokines as detailed in comprehensive maps of EGF receptor signaling [48]–[50].

In TCM or villous explant supernatants, HB-EGF was below the level of detection, and adding antagonists to HER1, HER2 and/or HER4 to migrating endometrial stromal cells did not diminish their response to TCM while effectively blocking that to isolated HB-EGF. Our data suggest that not HB-EGF but another as yet unidentified HER1 ligand is prominently present in TCM; yet this ligand does not exert a major contribution to the chemoattractant activity of TCM. It has been reported that the proteoglycan decorin binds to HER1 and evokes internalization and degradation of the receptor via caveolar-mediated endocytosis [51]. We therefore reasoned that decorin might be present in TCM. Western blot analyses, however, revealed decorin production and secretion by St-T1b cells and hESCs and up-regulation upon decidualization, but the proteoglycan was not detectable in trophoblast cell supernatants (data not shown).

HB-EGF has been recognized as a crucial mediator of embryo-uterine interactions in the mouse [32]. In humans, the growth factor is detected in uterine fluid aspirated immediately prior to embryo transfer in natural and stimulated cycles [52]. Endometrial expression peaks in the luminal and glandular epithelium at the time of implantation, while stromal expression decreases in the mid-secretory phase [53]–[57]. HB-EGF has been ascribed a role in promoting decidualization and survival of hESCs [44]. Although we did not detect HB-EGF secretion by the St-T1b endometrial stromal cell line, primary hESCs have been documented to produce HB-EGF in culture [44] and respond to the presence of a compromised embryo with decreased HB-EGF secretion [19]. In early pregnancy, HB-EGF is abundant in the decidua [58]. Our data suggest that local HB-EGF not only promotes invasion of trophoblast [29], [30] but may also participate in modulating endometrial stromal cell dynamics.

Our proteome profiling search for pro-migratory candidate factors in TCM and villous explant supernatants led to the identification of PDGF-AA, as mentioned above, and of VEGF and PLGF which both belong to the family of vascular endothelial growth factors. While the most common VEGF isoform, VEGF-A, binds to VEGF-R1 (FLT1) and, albeit with lower affinity, to VEGF-R2 (FLK1), PLGF selectively binds to VEGF-R1 [59]. VEGF-A and both receptor types are found in stromal, epithelial, endothelial and vascular smooth muscle cells of the endometrium throughout the cycle [59]. While VEGF-A is a certified stimulus of migration in various cell types including endothelial, mesenchymal and trophoblast cells [60]–[62], it did not elicit a migratory response of endometrial stromal cells in our study. This may partly be attributed to the fact that the cells themselves produced copious amounts of the factor, as revealed by proteome profiling. Moreover, VEGF action may be antagonized by the soluble form of VEGF-R1, sFLT1 [63]. PLGF has long been recognized as a prominent angiogenic trophoblast product [64]. Pertaining to the earliest stages of human pregnancy, PLGF mRNA was also detected in trophectoderm of day 5 blastocysts [43]. However, like VEGF, PLGF failed to stimulate endometrial stromal cell migration in our study. Again, this could be due to the production of saturating amounts of VEGF, or of antagonizing sFLT1 by the cultured cells. By RT-PCR, we detected transcripts for full length VEGF-R1 as well as two variants of sFLT1 (sFLT1-i13 and sFLT1-e15a) [65] in hESCs and St-T1b cells (data not shown), although we did not substantiate this at the protein level. *In vivo*, it will likely be the balance of pro-migratory VEGF and/or PLGF, and antagonizing sFLT1, and the presence of chemotactic gradients at the fetal-maternal interface, which will dictate extent and direction of migration in the numerous target cells.

Access to early placental tissues is limited, and procurement of sufficient numbers of purified trophoblast cells difficult as these primary cells rapidly cease to proliferate in culture. For large scale experiments, one therefore has to resort to cell lines with all inherent limitations [66]. The EVT-derived cell line AC-1M88 that we have chosen for our study has been designated an acceptable surrogate for primary cells for the study of adhesion, migration and invasion [67]. Our proteome profiling results further support the validity of this cell line. Almost all AC-1M88 secretory products were also present in supernatants of first trimester villous explant cultures. On the other hand, villous explants were found to produce a much broader spectrum of cytokines and angiogenesis factors, many of which could be attributed to the expression of the corresponding genes in either EVT or CTB. Additional sources may be the syncytiotrophoblast or the mesenchymal villous core. Although villous explant CM thus contained a much richer factor cocktail compared to TCM, it tended to be less chemoattractive to endometrial stromal cells. This may partly be due to the presence of inhibitory components like endostatin, ET-1, endoglin, TGF-β, PAIs and TIMPs (see Table 1) or additional inhibitory factors not covered by the spectrum of the proteome profiling arrays used in this study, including Dickkopf, Nodal or KISS10 [6].

In our attempt to dissect the signaling pathways relevant to random or chemotactic migration, we observed a reduction of basal chemotaxis (i.e. towards 10% steroid-depleted FCS in the lower compartment of the transwell chambers) upon inhibition of ERK1/2, p38 or PI3K signaling. The involvement of ERK1/2 and PI3K/Akt signaling in endometrial stromal cell chemotaxis, in response to PDGF-BB, has previously been reported [28]. No single inhibitor, however, markedly reduced migration towards TCM, suggesting the activation of multiple, partially redundant pathways by the trophoblast secretions. When assessing random migration in the presence of a uniform signal by means of the Oris migration assay (similar to the well-known scratch or wound healing assay), the PI3K pathway turned out to be essential for PDGF-BB stimulated motility.

Migration depends on cytoskeletal fluidity and continuous destabilization and stabilization of cortical actin stress fibers. ROCK1 activation by the Rho GTPase RhoA results in increased MLC phosphorylation, generating contractile forces through actin-myosin interactions [68]. Lamellipodial protrusions at the leading edge provide integrin-mediated adhesion to the underlying substrate. Contraction and detachment of trailing edges then allow promotion of the cell body [26]. Blocking Rho activity, which reduces ROCK1 activity, has been observed to increase lamellipodium extension, possibly by counteracting contraction [69]. In a coculture model of blastocysts on a monolayer of decidualized hESCs, motility of the stromal cells was found mandatory for blastocyst invasion. The outgrowth of blastocysts was enhanced by silencing of RhoA in the stromal cells, indicating an anti-invasive role of RhoA [24], [25]. Silencing of Raf-1, a serine/threonine kinase upstream of the MEK/ERK pathway [70], inhibits the migration of hESCs and coincides with increased ROCK activity, suggesting that excessive ROCK activity attenuates migration [71]. These studies fit well with our observation of enhanced endometrial stromal cell motility in the presence of ROCK inhibition, downstream of RhoA.

In our experiments, ROCK inhibition strikingly promoted random motility in the absence or presence of PDGF-BB. The ROCK inhibitor Y27632 also enhanced basal chemotaxis but did not further augment chemotaxis elicited by TCM. Interestingly, ROCK inhibition caused a rapid decrease in MLC2 phosphorylation. Likewise, decidualization concurred with sustained diminishment of total, and consequently, of phospho-MLC2. Increased phosphorylation of MLC, *vice versa*, prevents *in vitro* decidualization [72]. A negative role of elevated phospho-MLC2 in decidual cell migration is further supported by our observation that decidualized cells consistently displayed higher basal migration than did undifferentiated endometrial stromal cells.

With the exception of ROCK inhibitor, PDGF-BB was the only stimulus that activated stromal cell motility without providing directional information. PDGF-BB binding leads to PDGFRβ endocytosis and Rac1 activation at the cell membrane [73]. Because Rac1 antagonizes Rho activity [74], PDGF-BB could thus indirectly cause ROCK inhibition which contributes to enhanced motility. In terms of signaling activity, PDGF-BB stood apart from the chemotactic stimuli HB-EGF, PDGF-AA or TCM in its ability to cause sustained activation of Akt. This is in accordance with our finding that inhibition of the PI3K/Akt pathway was decisive in ablating chemokinesis.

The ability of decidual cells for random migration, in addition to directed movement towards trophoblast-derived signals, might aid in tissue remodeling at the implantation site. Decidualized hESCs produce MMPs and are invasive [21], [75] and could thus generate proteolytic tracks in the extracellular matrix to facilitate trophoblast invasion, analogous to fibroblast-led collective invasion of tumor cells [76].

In summary, our study described the function of PDGF-BB, HB-EGF and trophoblast-derived PDGF-AA in regulating endometrial stromal cell motility and provides further evidence for the active role of decidualized endometrial stromal cells in implantation and early pregnancy.

## Supporting Information

Figure S1 **Induction of decidualization markers in hESCs and St-T1b cells.** (**A**) Induction of transcripts for PRL, IGFBP-1 and FOXO1 upon decidualizing treatment (5d 8-Br-cAMP/MPA) was monitored by RT-PCR in two individual primary hESC cultures, and in the St-T1b human endometrial stromal cell line. (**B**) PRL and IGFBP-1 were measured by ELISA in culture supernatants after 5 or 7 d of decidualizing treatment. Secretion was normalized to RNA or protein content of the monolayer. Values are means±SD from 2–3 replicates. PRL secretion by St-T1b cells was mostly below the limit of detection (nd, not detectable). Methods: RNA was extracted and reverse-transcribed as detailed previously, and primer sequences and PCR conditions for amplification of transcripts for decidual PRL, IGFBP1, FOXO1 and GAPDH have been given elsewhere [33]. PCR products were resolved in 2% agarose gels and visualized by SYBR Gold staining (Molecular Probes/Life Technologies). PRL and IGFBP-1 secretion were assayed by ELISA kits from IBL International (Hamburg, Germany) and Mediagnost (Reutlingen, Germany), respectively, and normalized to total protein or RNA harvested from the underlying monolayer.
(TIF)

Figure S2 **Effect of pathway inhibitors on the appearance of hESCs in chemotactic migration assays.** Decidualized hESCs in transwell migration inserts were preincubated for 1 h with MEK1/2 inhibitor PD98059 (50 µM), ROCK inhibitor Y27632 (100 µM), Rac1 inhibitor NSC23766 (50 µM), p38 inhibitor SB202190 (10 µM) or PI3K inhibitor Wortmannin (200 nM) before the addition of trophoblast conditioned medium (TCM) to the lower reservoir for 18 h. Controls received MM1-10% instead of chemoattractant. Representative micrographs of migrated cells on the underside of the inserts are shown (Diff-Quik staining, 10× objective). The 8 µm pores in the membranes are seen as dots.
(TIF)

Figure S3 **Effect of pathway inhibitors on the appearance of St-T1b cells in chemokinetic migration.** Decidualized St-T1b cells were seeded at low density in chamber slides. Following 1 h preincubation with ROCK inhibitor Y27632 (100 µM) or PI3K inhibitor Wortmannin (200 nM), monolayers were treated with control medium or PDGF-BB (10 ng/ml). Eighteen hours later, cells were fixed and stained with Diff-Quik. Microphotographs were taken with a 20× objective. Note the extremely long protrusions formed in response to ROCK inhibition.
(TIF)

Protocol S1 **Quantification of migrated cells in the Oris^TM^ cell migration assay for chemokinesis.**
(PDF)
