# Supplementary figures and images for: Control of Human Endometrial Stromal Cell Motility by PDGF-BB, HB-EGF and Trophoblast-Secreted Factors
Source: PLoS One. 2013 Jan 21;8(1):e54336. doi: 10.1371/journal.pone.0054336 (PMC3549986; doi:10.1371/journal.pone.0054336)

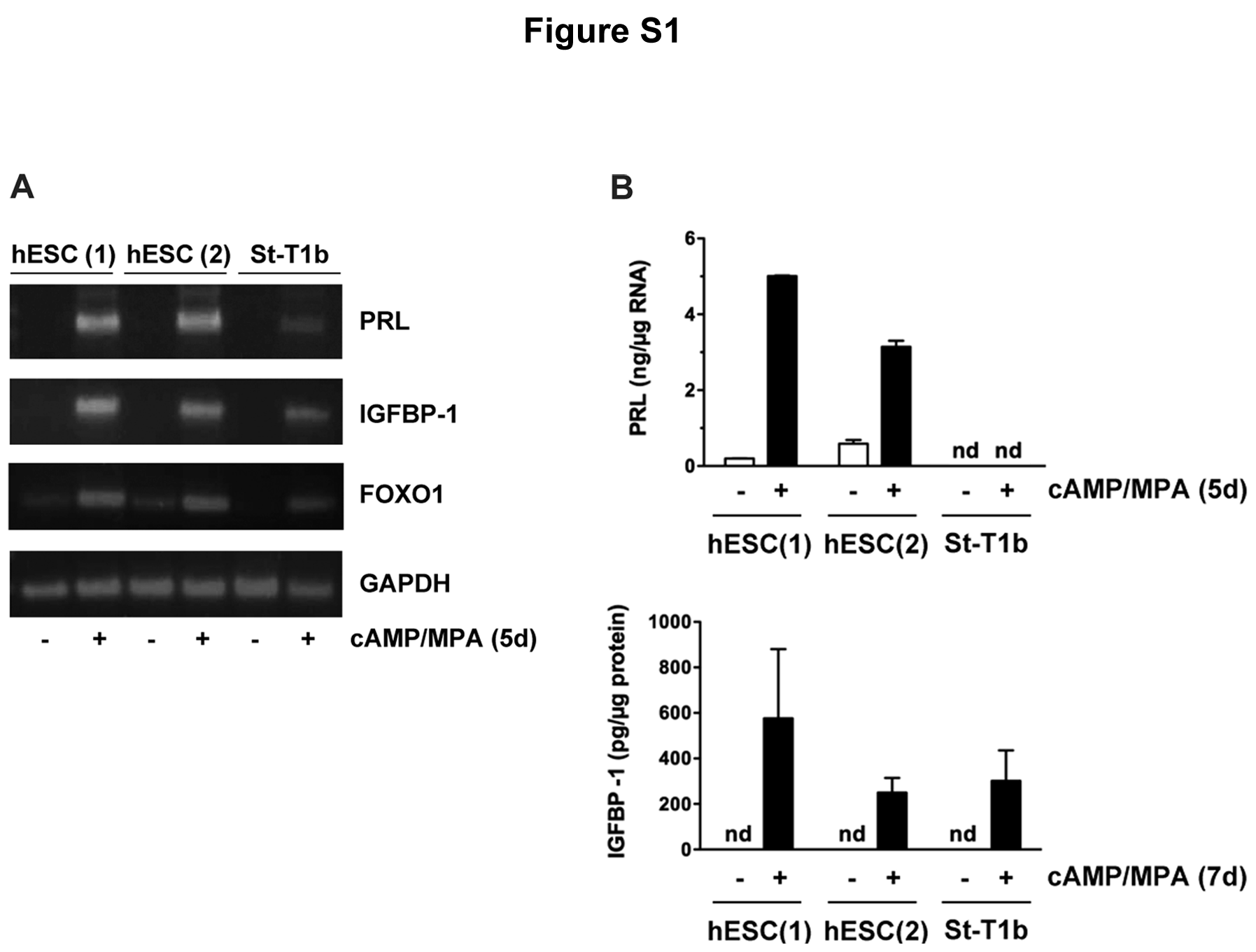

Supplement: Figure S1 — Induction of decidualization markers in hESCs and St-T1b cells. (A) Induction of transcripts for PRL, IGFBP-1 and FOXO1 upon decidualizing treatment (5d 8-Br-cAMP/MPA) was monitored by RT-PCR in two individual primary hESC cultures, and in the St-T1b human endometrial stromal cell line. (B) PRL and IGFBP-1 were measured by ELISA in culture supernatants after 5 or 7 d of decidualizing treatment. Secretion was normalized to RNA or protein content of the monolayer. Values are means±SD from 2–3 replicates. PRL secretion by St-T1b cells was mostly below the limit of detection (nd, not detectable). Methods: RNA was extracted and reverse-transcribed as detailed previously, and primer sequences and PCR conditions for amplification of transcripts for decidual PRL, IGFBP1, FOXO1 and GAPDH have been given elsewhere [33]. PCR products were resolved in 2% agarose gels and visualized by SYBR Gold staining (Molecular Probes/Life Technologies). PRL and IGFBP-1 secretion were assayed by ELISA kits from IBL International (Hamburg, Germany) and Mediagnost (Reutlingen, Germany), respectively, and normalized to total protein or RNA harvested from the underlying monolayer. (TIF) [file pone.0054336.s001.tif]

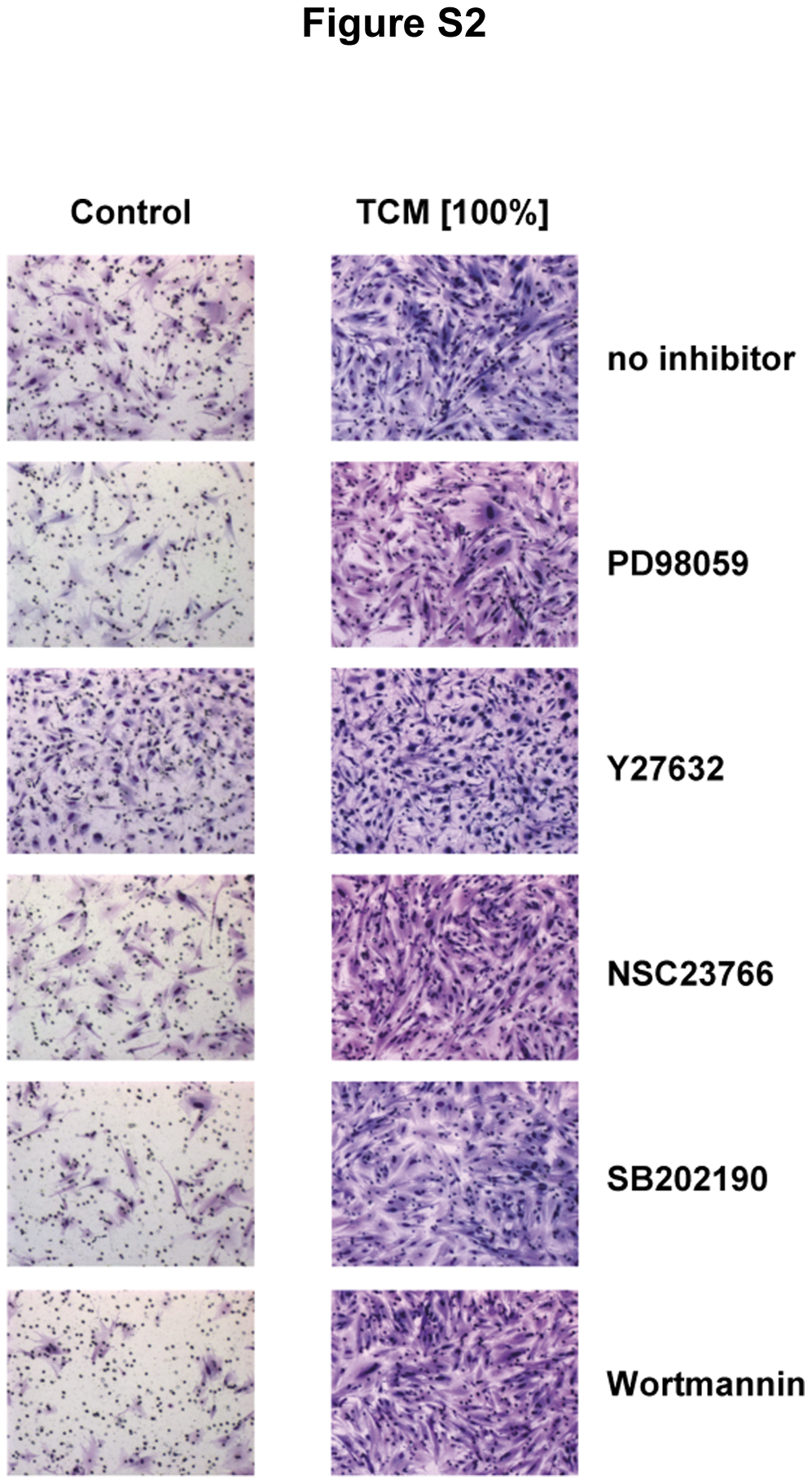

Supplement: Figure S2 — Effect of pathway inhibitors on the appearance of hESCs in chemotactic migration assays. Decidualized hESCs in transwell migration inserts were preincubated for 1 h with MEK1/2 inhibitor PD98059 (50 µM), ROCK inhibitor Y27632 (100 µM), Rac1 inhibitor NSC23766 (50 µM), p38 inhibitor SB202190 (10 µM) or PI3K inhibitor Wortmannin (200 nM) before the addition of trophoblast conditioned medium (TCM) to the lower reservoir for 18 h. Controls received MM1-10% instead of chemoattractant. Representative micrographs of migrated cells on the underside of the inserts are shown (Diff-Quik staining, 10× objective). The 8 µm pores in the membranes are seen as dots. (TIF) [file pone.0054336.s002.tif]

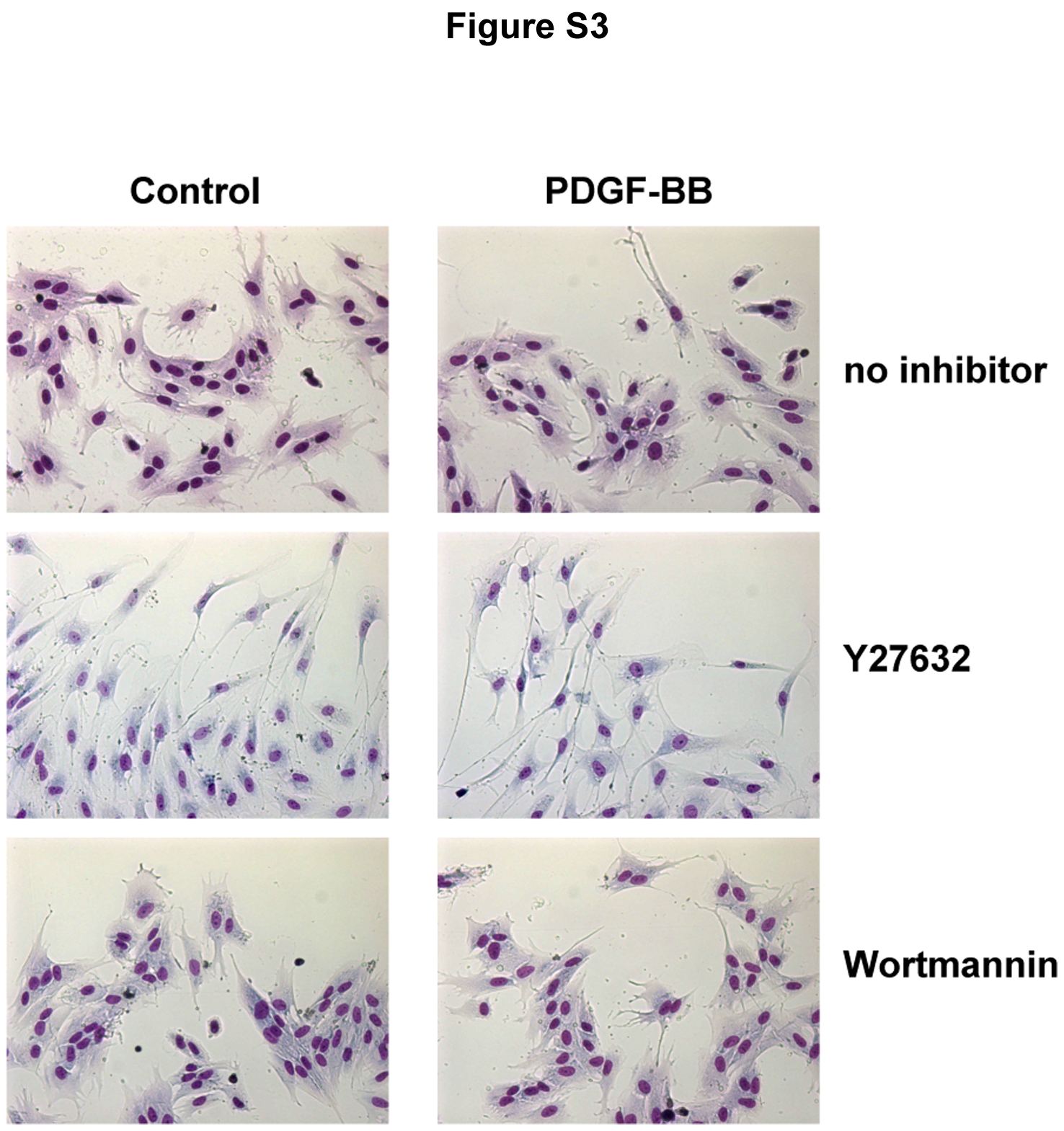

Supplement: Figure S3 — Effect of pathway inhibitors on the appearance of St-T1b cells in chemokinetic migration. Decidualized St-T1b cells were seeded at low density in chamber slides. Following 1 h preincubation with ROCK inhibitor Y27632 (100 µM) or PI3K inhibitor Wortmannin (200 nM), monolayers were treated with control medium or PDGF-BB (10 ng/ml). Eighteen hours later, cells were fixed and stained with Diff-Quik. Microphotographs were taken with a 20× objective. Note the extremely long protrusions formed in response to ROCK inhibition. (TIF) [file pone.0054336.s003.tif]
