# Supplementary material for: Control of Human Endometrial Stromal Cell Motility by PDGF-BB, HB-EGF and Trophoblast-Secreted Factors
Source: PLoS One. 2013 Jan 21;8(1):e54336. doi: 10.1371/journal.pone.0054336 (PMC3549986; doi:10.1371/journal.pone.0054336)
Supplement: Protocol S1 — Quantification of migrated cells in the OrisTM cell migration assay for chemokinesis. (PDF) [file pone.0054336.s004.pdf]

Protocol S1.

Quantification of migrated cells in the Oris™ cell migration assay for chemokinesis.

In the Oris™ cell migration assay, cells plated in black 96-well plates with clear flat bottoms are allowed to migrate into a central cell-free detection zone. According to the manufacturer's instructions, cells at the end of the migration period are stained with a fluorescent dye like Calcein AM, and a black masking plate is attached to the bottom of the 96-well plate. The masking plate has 96 prefabricated openings that are supposed to precisely frame the central detection zone of each well. Thus, the 96-well plate can be evaluated in a fluorescent microplate reader which will detect only signals resulting from cells that have migrated into the detection zone. We noted, however, that the stoppers are not consistently placed perfectly centrally in each well. The mask will then allow the shine-through of a lunate sickle-shaped part of the monolayer representing cells that have accumulated at the rim of the stopper, providing a strong fluorescent signal that obscures the signal resulting from cells that have actually migrated into the detection zone. We therefore modified the detection procedure as follows: after 18 h of migration, monolayers were fixed in methanol and stained with the Diff-Quik Staining Kit (Siemens Healthcare Diagnostics, Eschborn, Germany). Of each well, 4 overlapping microphotographs were acquired with a 4x objective in an inverted microscope (CKX41; Olympus, Hamburg, Germany) equipped with a cooled digital camera (CC-12; Olympus). Images covering the entire detection zone and part of the surrounding area were assembled from the microphotographs, and a virtual black mask (a graphic object with an opening representing the exact dimensions of the detection zone) was centered around the detection zone. The number of cells in the migration zone was determined using Fiji software (http://fiji.sc/wiki/index.php/Fiji). Briefly, images were converted to grey, and the threshold was adjusted such that the dark stained nuclei were highlighted as individual items which could be counted by the "analyze particles" function. In this way, numbers of migrated cells were determined from quadruplicate wells and the means normalized to control cells, having received MM1-10% only.
